# 14‐3‐3ζ targeting induced senescence in Hep‐2 laryngeal cancer cell through deneddylation of Cullin1 in the Skp1‐Cullin‐F‐box protein complex

**DOI:** 10.1111/cpr.12654

**Published:** 2019-06-21

**Authors:** Sung Bin Seo, Ji‐Ye Baek, Ji‐Hee Lim, Xuyan Jin, Mun‐Yong Lee, Jeong‐Hwa Lee

**Affiliations:** ^1^ Department of Biochemistry, College of Medicine The Catholic University of Korea Seoul South Korea; ^2^ Institute of Aging and Metabolic Diseases, College of Medicine The Catholic University of Korea Seoul South Korea; ^3^ Department of Biomedicine and Health Sciences, Graduate School, College of Medicine The Catholic University of Korea Seoul South Korea; ^4^ Division of Nephrology, Department of Internal Medicine, College of Medicine The Catholic University of Korea Seoul South Korea; ^5^ Department of Anatomy, College of Medicine The Catholic University of Korea Seoul South Korea

**Keywords:** 14‐3‐3 zeta, Cullin‐1, head and neck cancer, neddylation, senescence

## Abstract

**Objectives:**

Despite of the aberrant expression of 14‐3‐3ζ in head and neck squamous cell carcinoma (HNSCC), little is known about the role of 14‐3‐3ζ in the regulation of senescence in HNSCC. This study was performed to investigate whether 14‐3‐3ζ is implicated in senescence evasion of Hep‐2 laryngeal cancer cells.

**Methods:**

The expression of 14‐3‐3ζ was suppressed using RNA interference strategy. Senescence induction was determined by senescence‐associated β‐galactosidase staining and the numbers of promyelocytic leukaemia nuclear body. Real‐time PCR, western blotting and immunohistochemistry were applied for the expression of corresponding proteins. Xenograft experiment was performed to show in vivo effect of 14‐3‐3ζ silencing on tumour growth.

**Results:**

14‐3‐3ζ silencing significantly induced senescence phenotypes via 27 accumulations. Subsequently, we demonstrated that p27 accumulation is linked to inactivation of SCF^Skp2^ complex activity, probably due to the deneddylation of cullin‐1 (Cul‐1) as follows. (a) Neddylated Cul‐1 is decreased by 14‐3‐3ζ silencing. (b) Blocking neddylation using MLN4924 reproduces senescence phenotypes. (c) Knockdown of CSN5, which functions as a deneddylase, was shown to restore the senescence phenotypes induced by 14‐3‐3ζ depletion. Finally, we demonstrated that 14‐3‐3ζ depletion effectively hindered the proliferation of Hep‐2 cells implanted into nude mice.

**Conclusion:**

14‐3‐3ζ negatively regulates senescence in Hep‐2 cells, suggesting that 14‐3‐3ζ targeting may serve to suppress the expansion of laryngeal cancer via induction of senescence through the Cul‐1/SCF^Skp2^/p27 axis.

## 1 INTRODUCTION

Replicative senescence was first described as the state of permanent exit from cell cycle after a finite number of divisions.^1^ However, senescence can also be activated in replication‐competent cells in response to a variety of stressors, including oncogene activation, mitochondrial dysfunction, hypoxia and DNA damage. This form of cellular senescence, which occurs irrespective of the shortening of telomeres, is known as a premature senescence.^2^ In tumours, senescence represents a permanent loss of proliferation potential, which is thought to be a barrier for malignant transformation and expansion of tumour cells.^3^, ^4^ Chemotherapy and ionizing radiation can induce senescence‐like phenotypes in tumour cells and tissues that have already bypassed senescence, rendering senescence induction a promising tumour suppression strategy.^5^ Senescent cells exhibit several senescence‐associated phenotypes including an enlarged flattened morphology as well as upregulation of lysosomal β‐galactosidase activity that is responsible for the characteristic senescence‐associated β‐galactosidase (SA‐β‐gal) staining.^6^ At the molecular level, two critical tumour suppressor pathways driven by p16/Rb and p53/p21 play a crucial role in the induction and maintenance of the senescent state, an irreversible cell cycle arrest.^7^, ^8^ Therefore, frequent mutations in these pathways (p53 and p16) in tumours highlight the importance of senescence as a tumour suppression mechanism.^9^, ^10^, ^11^

Head and neck squamous cell carcinoma (HNSCC) arises in the oral cavity, larynx, pharynx and nasal cavity and is characterized by poor survival rates and a mortality rate of around 50%.^12^ The significant heterogeneity of its genomic and biological properties hampers the development of effective therapeutic strategy.^13^, ^14^ The idea of senescence as a tumour suppression strategy in HNSCC, as in other cancers, has been supported by several reports. The silencing of pro‐oncogenic risk factors of HNSCC such as MUC4 or FXR1 induces senescence in HNSCC cells through activation of the p16/Rb or p53/p21 pathway, respectively.^15^, ^16^ On the other hands, the re‐activation of tumour suppressor proteins including Myb‐binding protein 1A (MYPPB1A) or Fbxo4 as well as miRNAs such as miR‐34a or miR‐494‐3p which are suppressed in HNSCC tissues was shown to promote cell cycle arrest and senescence and enhance radiosensitivity in HNSCC cells.^17^, ^18^, ^19^, ^20^

Recently, 14‐3‐3ζ has been proposed as a potential oncogene involved in the pathogenesis of HNSCC. Namely, intense immunoreactivity for 14‐3‐3ζ was observed as early as in hyperplasia in oral pre‐malignant lesions, indicating that its overexpression is an early event in oral tumourigenesis.^21^ Moreover, its overexpression was found to be a strong predictor of poor prognosis of head and neck cancers in quantitative proteomics screens,^22^, ^23^ while suppression of 14‐3‐3ζ in tumour cells resulted in the induction of apoptosis and increased sensitivity to chemotherapeutics.^24^, ^25^ Furthermore, a recent report showed that 14‐3‐3ζ silencing retarded the proliferation and migration of tongue squamous cell carcinoma,^26^ suggesting that 14‐3‐3ζ may be serve as a target for effective inhibition of head and neck cancer progression. The pro‐survival activity of 14‐3‐3ζ might be primarily due to its ability of sequestering diverse pro‐apoptotic proteins such as BAD, BAX, ASK1, FOXO3 or c‐Abl in multiple type of cancer.^27^ However, little is known whether the oncogenic potential of 14‐3‐3ζ is associated with the inhibition of induction of cancer cell senescence. We have previously demonstrated that downregulation 14‐3‐3ζ or 14‐3‐3β led to stable cell cycle arrest of A172 and U87 glioblastoma cells, eventually leading to senescence and accumulation of p27.^28^, ^29^ It is thus probable that high expression of oncogenic 14‐3‐3 proteins in HNSCC allows the cancer cells to escape the senescence program, thereby promoting their oncogenic potential.

The progression of the cell cycle is governed by fine and timely modulation of the quantities of cell cycle regulators such as cyclin, cyclin‐dependent kinase (CDK) and CDK inhibitors. The precise regulation of cell cycle progression in normal cells is dependent on scheduled proteolytic degradation of regulatory proteins through the ubiquitin‐proteasome pathway. Thus, the disruption of regulated proteolytic pathways is closely associated with a permanent cell cycle arrest, which is the central feature of senescent cells. Two ubiquitin ligases, Skp1‐Cullin‐F‐box protein (SCF) complex and the anaphase‐promoting complex/cyclosome (APC/C), are mainly responsible for the specific ubiquitination and subsequent degradation of key regulators involved in cell cycle progression.^30^ Most SCF complexes comprise three invariable components Skp1 (adaptor protein), Cullin‐1 (Cul‐1, scaffold protein) and RBX (Ring finger protein) as well as a variable component F‐box protein, a receptor protein. Cul‐1 is a major structural scaffold involved in assembling the SCF complex. Cul‐1 interacts with Rbx1 and Skp1 via its C‐ and N‐terminals, respectively, recruiting Ub‐E2 to the SCF complex in close proximity to the substrates recognized by the F‐box protein.^31^, ^32^ To a great extent, the activity of SCF ubiquitin ligase is dependent on a covalent modification of Cul‐1, called neddylation.^33^, ^34^ The cullin family proteins are the best targets for neddylation, a type of posttranslational modification that conjugates NEDD8, an ubiquitin‐like molecule, to the target protein in three steps, which are similar to those of the ubiquitination process.^35^, ^36^ The neddylation of cullins retains the SCF complex in an active conformation, thus promoting the ubiquitination of diverse substrates involved in cell cycle progression, signal transduction and differentiation. Proteomic analysis has shown that 14‐3‐3ζ binds to Cul‐1^37^; however, the physiological significance of this interaction has not yet been clarified.

In the present study, we demonstrated that 14‐3‐3ζ knockdown causes cell cycle arrest, followed by senescence induction in Hep‐2 laryngeal cancer cells, via a p27‐dependent pathway. We also provide evidence that the neddylation status of Cul‐1 in the SCF complex is a critical determinant of p27 accumulation and premature senescence induced by 14‐3‐3ζ depletion in Hep‐2 cells. Taken together, these findings indicate that the induction of premature senescence through the suppression of 14‐3‐3ζ expression might be an effective therapeutic intervention for 14‐3‐3ζ‐overexpressing tumours.

## 2 MATERIALS AND METHODS

### 2.1 The cancer genome atlas and in silico analyses of 14‐3‐3ζ expression

The survival rates of 472 HNSCC patients in association with 14‐3‐3ζ expression levels were obtained from the cancer genome atlas (TCGA) dataset (https://cancergenome.nih.gov/). The patients were divided into two groups, the low and high expression of 14‐3‐3ζ, based on the best cut‐off FPKM (number Fragments Per Kilobase of exon per Million reads) values yielding the lowest log‐rank *P* value in the survival outcome (https://www.proteinatlas.org/about/assays). In silico analysis for 14‐3‐3ζ expression was obtained from the National Center for Biotechnology Information (NCBI) Gene Expression Omnibus (GEO) database portal (http://www.ncbi.nlm.nih.gov/geo/, Accession Number: GSE83519 and GSE51985). The relative expression of 14‐3‐3ζ in each datasets was determined by comparing the values in normal and tumour tissues.

### 2.2 Cell culture and transfection

Hep‐2 and SNU899 human laryngeal cancer cells were cultured in DMEM and RPMI 1640, respectively, supplemented with 10% FBS and 1% penicillin‐streptomycin (BioWest). MG132 and MLN4924 were purchased from Sigma‐Aldrich and Active Biochem, respectively. Suppression of 14‐3‐3ζ, p27 or Cdh1 expression was achieved by transfection with small interfering RNA (siRNA) using G‐fectin (Genolution). The specific sequences of siRNA used for the target genes are listed in Table S1.

### 2.3 Western blotting and immunoprecipitation

Western blotting and immunoprecipitation assays were conducted as described previously,^29^, ^38^ using the following antibodies: anti‐14‐3‐3ζ (Aviva Systems Biology Corporation), anti‐p27 (BD Bioscience), anti‐Skp2 (Cell Signaling), anti‐Cul‐1 (Invitrogen), anti‐Cdh1 (Abcam), anti‐p21, anti‐p16, anti‐CSN5 and anti‐β‐actin (Santa Cruz Biotechnology). Quantification of the intensities of bands was performed using imagej (NIH).

### 2.4 Quantitative real‐time PCR

Quantitative real‐time PCR (qRT‐PCR) was performed as previously described.^39^ The expression levels were normalized against the internal reference gene β‐actin, and relative expression levels were displayed using the ΔΔCt method. The specific primers for each mRNA are shown in Table S2.

### 2.5 SA‐β‐gal and immunofluorescence

Senescence‐associated β‐galactosidase staining was performed as described by previously.^6^ The percentage of SA‐β‐gal‐positive (blue‐stained) cells was measured from three randomly chosen fields under an inverted phase contrast microscope (Olympus). At least 100 cells were counted per experiment. The numbers of promyelocytic leukaemia nuclear body (PML‐NB) were determined by immunofluorescence analysis using antibodies specific for PML (Santa Cruz Biotechnology) under a Leica DMi8 microscope^40^ (Leica).

### 2.6 Cell growth and cell cycle analysis

Cell numbers at the indicated days were determined with hemocytometer after trypan blue staining. For colony‐forming assay, cells were re‐seeded into 6‐well plates at the density of 1000 cells/well after 24 hours of transfection with 14‐3‐3ζ siRNA. The colony numbers were determined by 0.2% crystal violet staining after 14 days of culture. Cell cycle distribution was analysed through DNA content staining using propidium iodide (50 μg/mL) and RNase A (1 mg/mL; Sigma‐Aldrich). Flow cytometry (FACSCanto; BD Bioscience) data acquisition and analysis were performed using flow jo software (FlowJo).

### 2.7 Mouse tumour models

Animal studies were approved by the Institutional Animal Care and Use Committee at Catholic University of Korea. Hep‐2 cells were pre‐treated with control or 14‐3‐3ζ siRNA for 48 hours, after which 1 × 10^7^ cells in 200 μL PBS were injected subcutaneously into the flanks of 5‐6‐week‐old male BALB/c nude mice (Orient bio Inc). Two weeks after tumour cell inoculation, all mice were sacrificed, and individual tumours were weighted and fixed in 4% paraformaldehyde and embedded in paraffin or frozen in Tissue‐Tek optimum cutting temperature (Sakura Finetek). Staining for 14‐3‐3ζ and p27 was carried out on paraffinized sections.

### 2.8 Statistics

Data are expressed as mean values ± SEM. Comparison between two different groups was assessed by Student's *t* test. *P *< 0.05 was considered statistically significant.

## 3 RESULTS

### 3.1 Silencing 14‐3‐3ζ induces growth retardation and premature cellular senescence in Hep‐2 human laryngeal cancer cells

**Figure 1:**
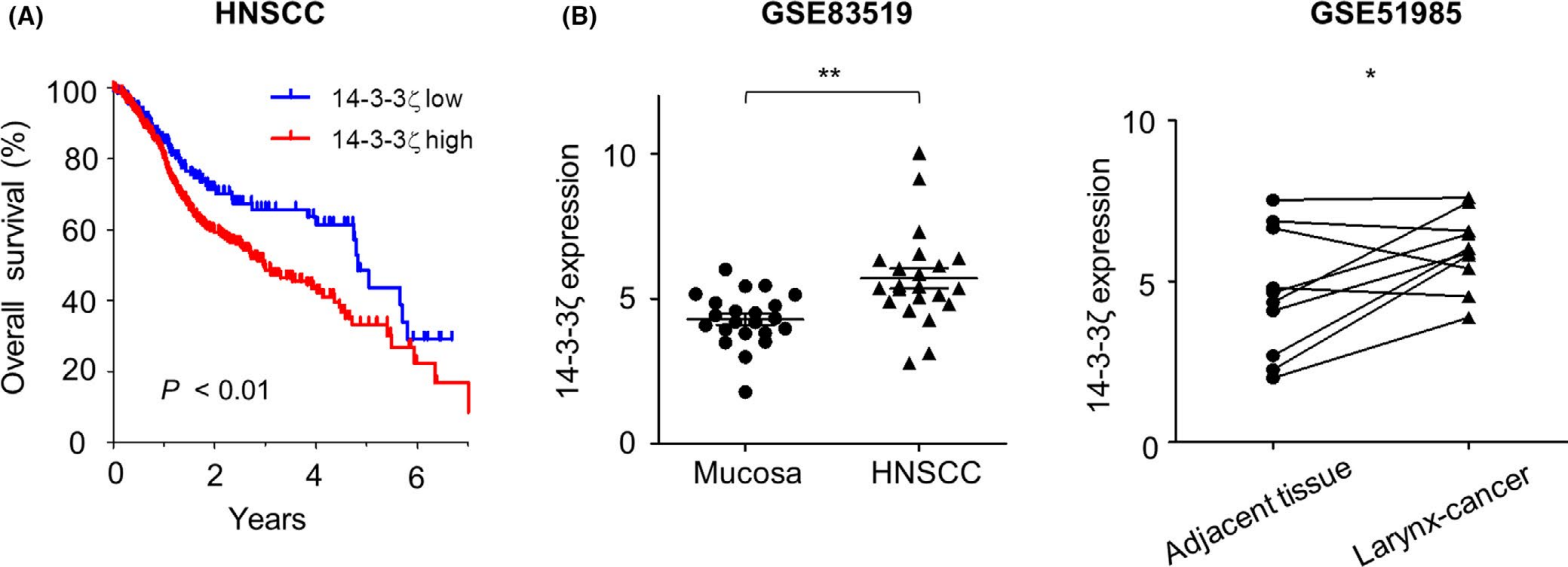
High expression of 14‐3‐3ζ in head and neck squamous cell carcinoma (HNSCC) and laryngeal cancers. A, Overall survival of 335 patients with high 14‐3‐3ζ expression and 137 patients with low expression (*P *<0.01). B, The relative level of 14‐3‐3ζ was analysed in HNSCC and corresponding normal mucosa (GSE83519, N = 22) and in laryngeal cancer which paired with normal adjacent tissues (GSE51985, N = 10). *P < 0.05, **P < 0.01

**Figure 2:**
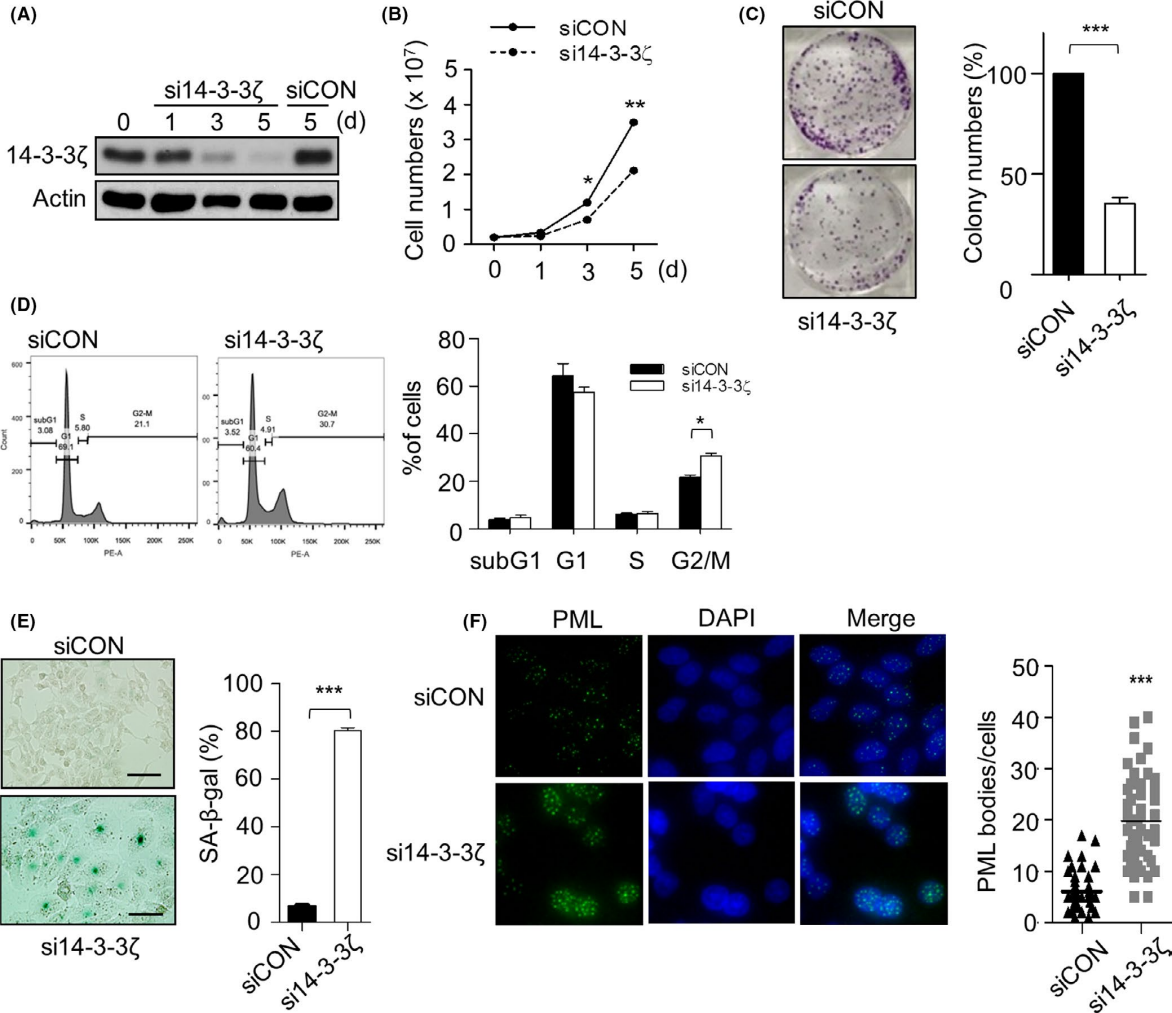
Effect of the depletion of 14‐3‐3ζ on cell growth and morphological changes in Hep‐2 cells. Hep‐2 cells were treated with 50 nmol/L of 14‐3‐3ζ siRNA (si14‐3‐3ζ) or control siRNA (siCON) for the indicated duration. d, day. A, The suppression of 14‐3‐3ζ expression was confirmed by western blotting. β‐Actin serves as a loading control. B, The effect of 14‐3‐3ζ depletion on cell growth was assessed by determining the cell numbers using trypan blue exclusion assay. C, Colony numbers were counted at 14 d after transfection with si14‐3‐3ζ and presented as percentages of colony numbers in the control. D, Hep‐2 cells were stained with propidium iodide, and the distribution of cells in each cell phase was analysed by flow cytometry at 5 d following transfection of each siRNA. The representative (left) and quantitative results (right) are shown. E, The effect of 14‐3‐3ζ depletion on the induction of senescence was observed by senescence‐associated β‐galactosidase (SA‐β‐gal) staining (left) and presented as the percentage (%) of SA‐β‐gal‐positive cells (right). Scale bar, 50 µm. F, Promyelocytic leukaemia (PML) bodies were stained by immunofluorescence (left) and are shown as the number of PML bodies per cell for each treatment (right). Bar indicates the mean value from fifty cells. Data (A‐E) represent the mean value ± SEM from at least three independent experiments. *P < 0.05, **P < 0.01, ***P < 0.001

The clinical significance of 14‐3‐3ζ in HNSCC was assessed by TCGA analysis indicating that 14‐3‐3ζ expression level is associated with survival rates of 472 HNSCC patients (Figure 1A). In silico analysis using two GEO data sets demonstrated that 14‐3‐3ζ expression level is higher at tumour site in HNSCC as well as in laryngeal cancer (Figure 1B). To investigate whether 14‐3‐3ζ targeting can inhibit the expansion of laryngeal cancers, we examined the proliferation and morphological change of Hep‐2 laryngeal cancer cells following suppression of 14‐3‐3ζ expression using RNA interference (Figure 2A). The depletion of 14‐3‐3ζ expression significantly retarded the cell proliferation rate of Hep‐2 cells, as determined by cell number counting (Figure 2B). Furthermore, 14‐3‐3ζ knockdown suppressed the colony‐forming ability of Hep‐2 cells to 35.4% of that of control cells (Figure 2C). Cell cycle analysis indicated that 14‐3‐3ζ depletion increased the G2/M population compared to control, siRNA‐transfected cells, while no significant increase in the sub‐G1 proportion was observed, indicating that it was not the induction of apoptosis, but cell cycle retardation, that was associated with the inhibition of 14‐3‐3ζ‐depleted Hep‐2 cell growth (Figure 2D). In the morphological aspect, Hep‐2 cells treated with 14‐3‐3ζ siRNA were large and of a flattened shape, a typical finding of senescent cells. Moreover, the extent of SA‐β‐gal staining increased to 80.43% in the 14‐3‐3ζ‐silenced cells (Figure 2E). We also observed the induction of senescence by 14‐3‐3ζ silencing in another laryngeal cancer cell line, SNU899, as determined by the increase in the SA‐β‐gal‐positive population (Figure S1). Moreover, the number of PML‐NB per cell, an additional senescence marker, increased 3.33‐fold after 14‐3‐3ζ silencing (Figure 2F). Collectively, these results indicate that 14‐3‐3ζ depletion significantly suppressed the proliferation of Hep‐2 cells via the induction of premature senescence.

### 3.2 Senescence induced by 14‐3‐3ζ depletion is attributable to the accumulation of p27

**Figure 3:**
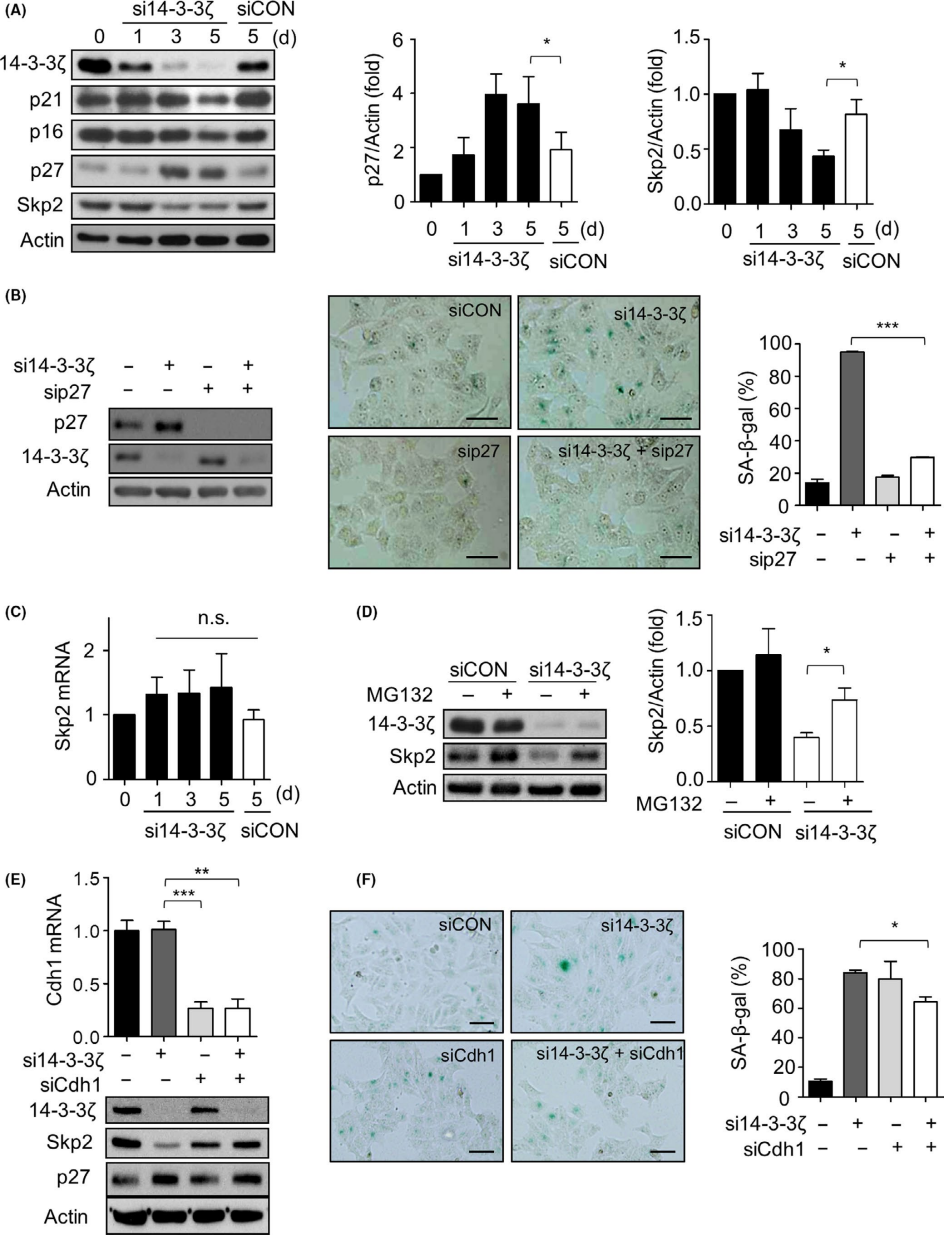
14‐3‐3ζ silencing‐mediated senescence is dependent on p27 accumulation but not on Skp2 levels. A, The expression levels of 14‐3‐3ζ, p21, p16, Skp2 and p27 were detected by western blotting following transfection with 50 nmol/L of siCON or si14‐3‐3ζ (left). Densitometric analysis of p27 (middle) and Skp2 (right) protein levels from three independent experiments. The value normalized against Actin at day 0 is designated as 1.0. B, Co‐transfection of p27 siRNA (sip27, 50 nmol/L) restored the senescence induced by 14‐3‐3ζ depletion, as determined by senescence‐associated β‐galactosidase (SA‐β‐gal) staining (middle) and the percentage (%) of SA‐β‐gal‐positive cells (right). Western blotting confirmed the effective suppression of 14‐3‐3ζ and p27 (left). Scale bar, 50 µm. C, *Skp2* mRNA levels were examined by quantitative real‐time PCR. n.s, not significant. D, Hep‐2 cells were transfected with siCON or si14‐3‐3ζ for 2 d followed by treatment with 10 μmol/L MG132 for 4 h, and then Skp2 levels were determined by western blotting (left). The recovery of Skp2 levels was evaluated by densitometric analysis from three independent experiments (right). E, F, Hep‐2 cells were transfected with si14‐3‐3ζ with/without Cdh1 siRNA (siCdh1) (50 nmol/L) for 4 d. Cdh1 suppression was confirmed by qRT‐PCR (E, upper panel). The expression levels of 14‐3‐3ζ, Skp2 and p27 in each treatment were examined by western blotting (E, lower panel). Representative image of SA‐β‐gal staining assay was presented (F, left). Percentage of SA‐β‐gal‐positive cells (F, right) in each group indicates that suppression of Cdh1 expression did not prevent 14‐3‐3ζ depletion‐induced senescence. Scale bar, 100 µm. Data represent mean values ± SEM of three independent experiments. Scale bar, 100 µm. **P < 0.01, ***P < 0.001 between indicated groups

To further evaluate the molecular basis of senescence driven by 14‐3‐3ζ depletion, we examined the expression of cell cycle inhibitory regulators p21, p16 and p27. Interestingly, the p27 expression levels increased in a time‐dependent manner, while no detectable changes were observed in p21 and p16 levels (Figure 3A). We additionally validated the importance of accumulation of p27 in 14‐3‐3ζ depletion‐induced senescence using co‐transfection of 14‐3‐3ζ and p27 siRNAs. We observed that the senescence‐like morphology was reversed by p27 knockdown in 14‐3‐3ζ‐depleted cells from 95% to 29%, as measured by the proportion of SA‐β‐gal‐positive cells (Figure 3B). Figure 3A also showed that the expression of Skp2, a major determinant for p27 levels,^41^ gradually decreased to 50% of control levels at day 5 after 14‐3‐3ζ silencing. However, *Skp2* mRNA levels did not parallel with Skp2 protein levels (Figure 3C), suggesting that 14‐3‐3ζ is involved in the post‐transcriptional regulation of Skp2 turnover in Hep‐2 cells. Our presumption is supported by subsequent experiments showing that treatment with the proteasome inhibitor MG132 increased Skp2 levels by about 2‐folds compared to those in cells treated only with 14‐3‐3ζ siRNA (Figure 3D). The degradation of Skp2 is mainly mediated by the ubiquitin ligase APC/C, which contains Cdh1 as a co‐activator.^42^, ^43^ Silencing of Cdh1 resulted in the restoration of Skp2 levels in 14‐3‐3ζ‐depleted cells (Figure 3E). However, neither p27 accumulation nor SA‐β‐gal‐positive cells were reversed by simultaneous silencing of Cdh1 and 14‐3‐3ζ (Figure 3E,F). These results indicate that the reduction in Skp2 is not directly involved in p27 accumulation and subsequent induction of premature senescence induced by 14‐3‐3ζ silencing in Hep‐2 cells.

### 3.3 Attenuated neddylation of Cul‐1 is the critical determinant for driving the senescence pathway following 14‐3‐3ζ silencing

**Figure 4:**
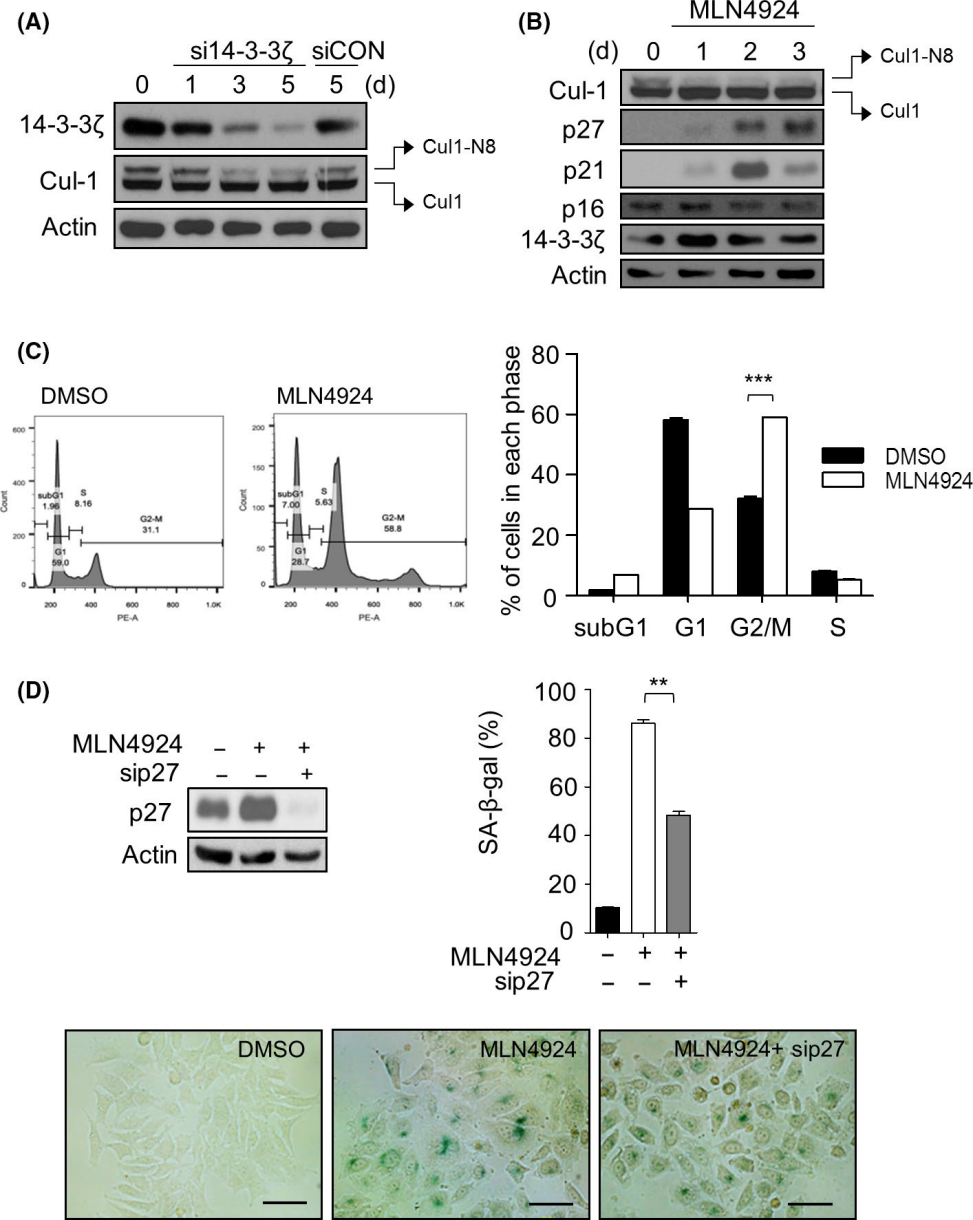
14‐3‐3ζ silencing‐mediated senescence is associated with deneddylation of Cul‐1. A, Following 14‐3‐3ζ siRNA treatment, the neddylation status of Cul‐1 was determined by western blotting. Neddylated Cul‐1 was indicated as Cul‐1‐N8. B, Hep‐2 cells were treated with 1 μmol/L of MLN4924 for 3 d. Total protein lysates were subjected to western blotting to assess the levels of Cul‐1, Skp2, p27, p21, p16 and 14‐3‐3ζ, with β‐Actin used as loading control. C, Hep‐2 cell was treated with MLN4924 for 48 h, after which cells were collected, and cell cycle was analysed by flow cytometry (left). The percentage of cells in each phase was obtained using flowjo software (right). D, Silencing of p27 partially blocks the induction of senescence by MLN4924. The expression levels of p27 were examined by western blotting (left). The percentage (%) of senescence‐associated β‐galactosidase (SA‐β‐gal)‐positive cells (right) and the representative pictures of SA‐β‐gal staining (lower) in each group are shown. Scale bar, 50 µm. **P < 0.01, ***P < 0.001, between indicated groups. Values are mean values ± SEM of triplicate experiments

To determine whether the decrease in SCF ubiquitin ligase activity is responsible for p27 accumulation in 14‐3‐3ζ‐depleted Hep‐2 cells, we examined the neddylation status of Cul‐1, which is required for SCF–Skp2 activity.^33^ Figure 4A shows that neddylated Cul‐1 (Cul1‐N8) levels decreased after 14‐3‐3ζ depletion in a time‐dependent manner. Hep‐2 cells were then exposed to the NEDD8‐activating enzyme 1 (NAE1) inhibitor MLN4924^44^, ^45^ to block the neddylation process in order to investigate whether MLN4924 can reproduce the senescence‐inducing effect of 14‐3‐3ζ silencing in Hep‐2 cells. Treatment of MLN4924 resulted in a gradual decrease in neddylated Cul‐1 levels and a concomitant increase in p27 and p21 levels, as well as a profound arrest of the cell cycle in the G2 phase (Figure 4B,C). After MLN4924 treatment, the proportion of SA‐β‐gal‐positive cells increased to 86.12%, which was reduced to 48.4% by co‐transfection with p27 siRNA (Figure 4D). The extent of the recovery effect of p27 silencing on MLN4924‐induced senescence being lesser than in 14‐3‐3ζ‐depleted cells (Figure 2E) indicated that p27 contributes substantially, if not exclusively, to MLN4924‐mediated senescence in Hep‐2 cells. The remaining proportion may be due to the general inhibitory effect of MLN4924 on most of the proteins of the cullin family, resulting in accumulation of other substrates such as p21. Taken together, these results indicate that p27 accumulation and senescence induced by depletion of 14‐3‐3ζ are dependent on neddylation of Cul‐1.

**Figure 5:**
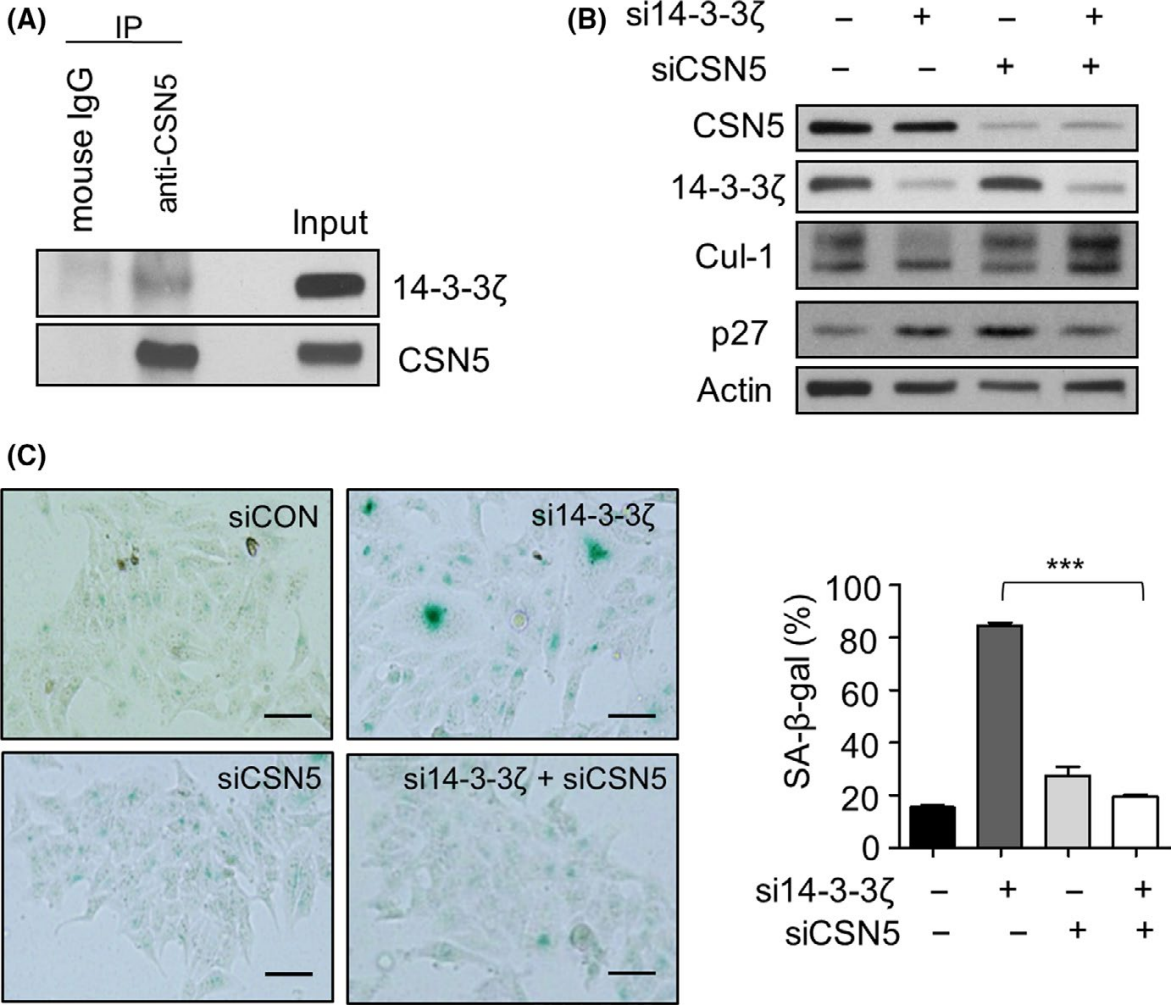
Suppression of CSN5 restored the senescence phenotypes induced by 14‐3‐3ζ depletion. A, The interaction of 14‐3‐3ζ and CSN5 was verified by co‐immunoprecipitation analysis. Total lysates were immunoprecipitated with anti‐CSN5 antibody (IP), followed by immunoblotting with antibodies for 14‐3‐3ζ or CSN5. (B) Hep‐2 cells were transfected with 50 nmol/L CSN5 siRNA (siCSN5) and/or si14‐3‐3ζ, and western blotting was performed with the indicated antibodies. C, The effect of CSN5 silencing on the senescence induced by 14‐3‐3ζ depletion was verified by senescence‐associated β‐galactosidase (SA‐β‐gal) staining (left) and presented as percentage (%) of SA‐β‐gal‐positive cells (right). Scale bars, 100 μm. ***P < 0.001 between indicated groups

Previously, systematic quantitative proteomics revealed the interaction of Cul‐1 and 14‐3‐3ζ.^37^ Considering that 14‐3‐3ζ is a scaffold protein lacking enzymatic activity, it is not likely that 14‐3‐3ζ directly affects the neddylation of Cul‐1. In addition, 14‐3‐3ζ was shown to bind to the 5th component of the COP9 signalosome (CSN5), a proteolytic subunit of CSN, which catalyzes the deneddylation process for disassembly and remodelling of the SCF complex.^46^, ^47^, ^48^ Therefore, it is possible that 14‐3‐3ζ regulates the neddylation of Cul‐1 through CSN5. To test this hypothesis, we first performed co‐immunoprecipitation to confirm the interaction of CSN5 and 14‐3‐3ζ: 14‐3‐3ζ was detected in the immune complex precipitated with the antibody against CSN5 (Figure 5A). We next co‐transfected Hep‐2 cells with CSN5 and 14‐3‐3ζ siRNAs to investigate whether the inhibition of CSN5 expression affects the induction of senescence by 14‐3‐3ζ silencing. Immunoblotting showed that neddylated Cul‐1 was increased while p27 level was decreased by co‐transfection with CSN5 and 14‐3‐3ζ siRNA (Figure 5B, lanes 2 and 4). Subsequently, we found that the senescence‐like morphology and increase of SA‐β‐gal‐positive cells induced by 14‐3‐3ζ depletion were rescued from 84.4% to 19.6% by CSN5 knockdown (Figure 5C). Based on these observations, 14‐3‐3ζ might prevent CSN5 function, probably through physical interaction, and depletion of 14‐3‐3ζ might release CSN5 to function as deneddylase, resulting in inactivation of the SCF complex.

### 3.4 14‐3‐3ζ depletion suppresses the growth of Hep‐2 cell xenografts

**Figure 6:**
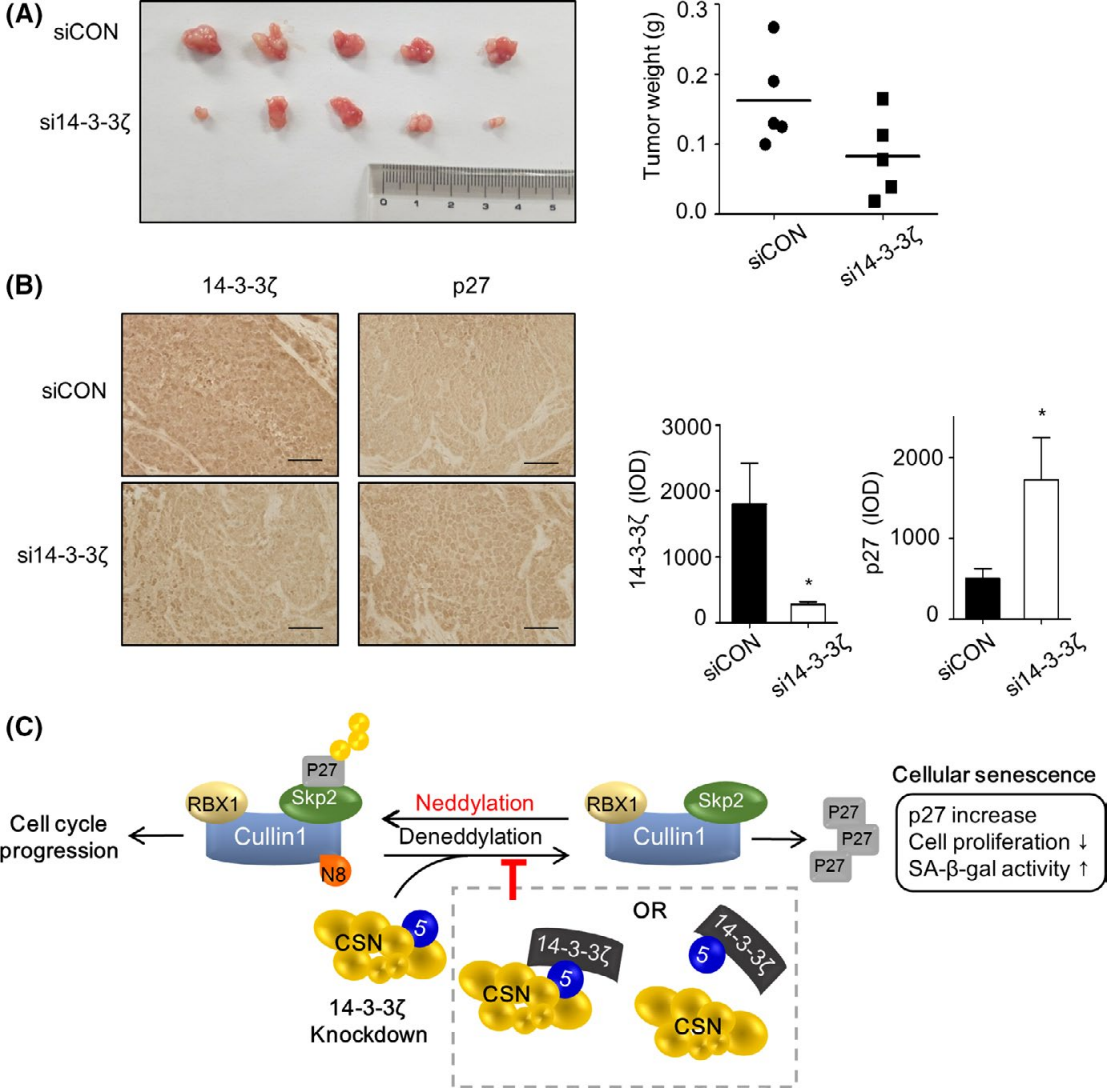
14‐3‐3ζ depletion retarded tumour growth in mouse xenograft. Hep‐2 cells transfected with control or 14‐3‐3ζ siRNA for 48 h and then injected into nude mice. A, Tumour weight in each group xenograft mice was measured at 2 weeks after injection. The weight range was indicated as graph (right, n = 5, *P *= 0.08). B, Levels of 14‐3‐3ζ and p27 were examined by immunohistochemistry. Representative images are shown at left panels, and the quantitation results were provided as integrated optical intensities (IOD, right panels). *P < 0.05. Scale bar, 100 μm. C, A proposed model for the molecular axis which is involved in the senescence induction by 14‐3‐3ζ silencing

Our in vitro data indicated that 14‐3‐3ζ depletion‐mediated senescence could efficiently suppress the expansion of Hep‐2 cells. To test whether 14‐3‐3ζ silencing can inhibit tumour growth in vivo, Hep‐2 cells, which were treated with control or 14‐3‐3ζ siRNA for 2 days, were injected into nude mice subcutaneously. Depletion of 14‐3‐3ζ significantly reduced the tumour mass after 14 days of injection (Figure 6A). The mean tumour weight from the control and the 14‐3‐3ζ siRNA‐treated Hep‐2 cells was 0.16 and 0.08 g, respectively (*P *= 0.08). Immunostaining of 14‐3‐3ζ and p27 showed that the decreased expression of 14‐3‐3ζ and increased expression of p27 were sustained in the tumour mass derived from 14‐3‐3ζ siRNA‐treated Hep‐2 cells (Figure 6B). These results indicate that 14‐3‐3ζ plays a critical oncogenic role in Hep‐2 cells and that depletion of 14‐3‐3ζ inhibits proliferation of Hep‐2 cells, probably via induction of senescence, both in vitro and in vivo.

## 4 DISCUSSION

Head and neck squamous cell carcinoma is the sixth leading cancer by incidence worldwide. It is genetically heterogeneous and biologically aggressive in nature, which leads to poor prognosis, despite advancements in standard multimodal therapies.^12^, ^14^ Therefore, identification of an effective molecular target is necessary to delay its progression. Despite the increasing body of evidence suggesting a strong link between 14‐3‐3ζ and HNSCC, the effect of 14‐3‐3ζ on the regulation of senescence in HNSCC has not been extensively studied. In the present study, we demonstrated that suppression of 14‐3‐3ζ expression strongly retarded the growth of Hep‐2 laryngeal cancer cells, concomitant with the occurrence of the senescence phenotype, as evidenced by an increase in senescence‐specific SA‐β‐gal staining‐positive cells and by accumulation of PML‐NB (Figure 2E,F). Our results also showed that 14‐3‐3ζ silencing‐mediated senescence is dependent on p27, as shown by the recovery of Hep‐2 cells from senescence by p27 silencing (Figure 3B). These findings are consistent with our previous observation that premature senescence in glioblastoma is induced by 14‐3‐3ζ or 14‐3‐3β silencing, which is also accompanied by p27 accumulation, without affecting p16 or p21 expression.^28^, ^29^ The p16/pRB and p53/p21 axes are two major senescence‐triggering pathways that are activated in response to various stressors.^49^ However, p53 is frequently inactivated by mutation or alternatively by HPV oncogenes in more than 50% and 20% cases of HNSCC, respectively.^13^ The pRB/p16 pathway is also suppressed by HPV, mutation of CDKN2A or overexpression of cyclin D1 in HNSCC,^14^ which might increase the chances for tumours to evade senescence, leading to unlimited proliferation. Therefore, targeting 14‐3‐3 proteins presents itself as a potent strategy of suppressing HNSCC through the acceleration of p27‐dependent senescence.

The expression of p27 is mainly regulated by proteasomal degradation mediated by the SCF‐ ubiquitin ligase complex, which includes Skp2 for specific recognition of p27 as a substrate.^41^, ^50^ In our previous studies in glioblastoma cells, p27 accumulation following depletion of 14‐3‐3ζ or β was attributable to the decrease in *Skp2* mRNA through the loss of STAT3 or ERK activity following 14‐3‐3ζ or β depletion, respectively.^28^, ^29^ However, the increase in Skp2 protein levels was not the primary cause of p27 accumulation in the present study (Figure 3E,F). Therefore, even though p27 accumulation is the critical requirement both in 14‐3‐3ζ and β depletion‐mediated senescence in glioblastoma and Hep‐2 cells, the signalling axis upstream of p27 is not shared by 14‐3‐3β and ζ, nor by glioblastoma and laryngeal cancer.

Our subsequent analyses revealed that deneddylation of Cul‐1, a component of the SCF^Skp2^ complex, is responsible for inducing the senescence of Hep‐2 cells after 14‐3‐3ζ silencing (Figures 4 and 5). First, neddylated Cul‐1 levels are decreased by 14‐3‐3ζ depletion. Second, treatment with MLN4924, which blocks the neddylation of Cul‐1, reproduced the senescence phenotype and p27 accumulation. Third, co‐immunoprecipitation assays revealed that 14‐3‐3ζ interacts with CSN5, which is involved in the deneddylation process. Fourth, 14‐3‐3ζ depletion‐induced p27 accumulation and increase in the proportion of SA‐β‐gal‐positive cells were rescued by knockdown of CSN5. Based on these findings, we propose a signalling axis connecting 14‐3‐3ζ and senescence: the depletion of 14‐3‐3ζ expression accelerates the deneddylation of Cul‐1 and subsequent decrease in SCF^Skp2 ^activity, resulting in p27 accumulation and senescence of Hep‐2 cells (Figure 6C). To the best of our knowledge, this is the first report on the regulation of the neddylation pathway of SCF^Skp2^ by a 14‐3‐3 protein. At the present, however, it is not certain how 14‐3‐3ζ regulates the neddylation of Cul‐1. Considering the binding ability of 14‐3‐3ζ to CSN5 in the previous studies as well as the present study, it is possible that 14‐3‐3ζ inhibits the deneddylase activity of CSN by binding to CSN5 in the CSN complex. CSN5 can also exist in a free form outside the CSN complex both in the cytoplasm and nucleus whereas CSN‐associated CSN5 is located primarily in the nucleus.^51^, ^52^ Thus, another probable explanation is that the free form of CSN5 is sequestered by 14‐3‐3ζ and is unable to bind to the CSN complex, thereby allowing the neddylation status of Cul‐1, an active conformation of the SCF complex. The latter possibility was supported by our experiments, which showed that depletion of 14‐3‐3ζ increased the CSN5 expression in the nucleus fraction while decreased in the cytoplasmic fraction (Figure S2). This finding suggests that 14‐3‐3ζ may regulate translocation of CSN5 via the interaction, and the subsequent deneddylation pathway of Cul‐1. However, the free form of CSN5 was shown to specifically interact with p27, resulting in the nuclear export and subsequent degradation of p27.^53^ Therefore, the possibility that 14‐3‐3ζ may affect the translocation of CSN5 for p27 turnover, which does not involve activity of the SCF complex, should not be excluded.

In addition to the in vitro study, we also demonstrated that 14‐3‐3ζ silencing effectively inhibited the growth of tumour cells derived from Hep‐2 cells in a mouse xenograft tumour model (Figure 6), suggesting that 14‐3‐3ζ targeting may be promising strategy for restricting the expansion of HNSCC in vivo. With regard to targeting 14‐3‐3ζ proteins, several approaches, including peptide inhibitors and RNA interference, both natural and synthetic, are under experimental conditions. However, in vivo deliverability, off target effects of RNA interference and/or lack of isoform specificity are limiting factors.^54^, ^55^ Furthermore, 14‐3‐3ζ targeting is likely to have unknown consequences even in normal cells due to its abundant expression in normal cells.^55^ Therefore, a more detailed study on the molecular basis that links 14‐3‐3ζ and the downstream pathway such as Cul‐1 neddylation should be conducted in order to develop specific inhibitors that interfere with the interaction of 14‐3‐3ζ and critical target proteins, instead of targeting 14‐3‐3ζ directly.

In summary, we have demonstrated that 14‐3‐3ζ silencing significantly induces premature senescence in Hep‐2 laryngeal cancer cells, concomitantly upregulating p27, which is driven from the inactivation of the SCF ubiquitin ligase through the deneddylation of Cul‐1. These findings provide a novel insight into the 14‐3‐3ζ–Cul‐1‐p27 axis as a potential therapeutic target for the treatment of HNSCC.

## CONFLICT OF INTEREST

We declare that all the authors have no conflict of interest.

## AUTHOR CONTRIBUTION

SS and JH Lee conceived the study. SS, JYB, JH Lim, XJ performed the experiments. MYL and JH Lee analysed the data. SS and JH Lee wrote the manuscript. All authors read and approved the final manuscript.
