# Supplementary figures and images for: 14‐3‐3ζ targeting induced senescence in Hep‐2 laryngeal cancer cell through deneddylation of Cullin1 in the Skp1‐Cullin‐F‐box protein complex
Source: Cell Prolif. 2019 Jun 21;52(5):e12654. doi: 10.1111/cpr.12654 (PMC6797561; doi:10.1111/cpr.12654)

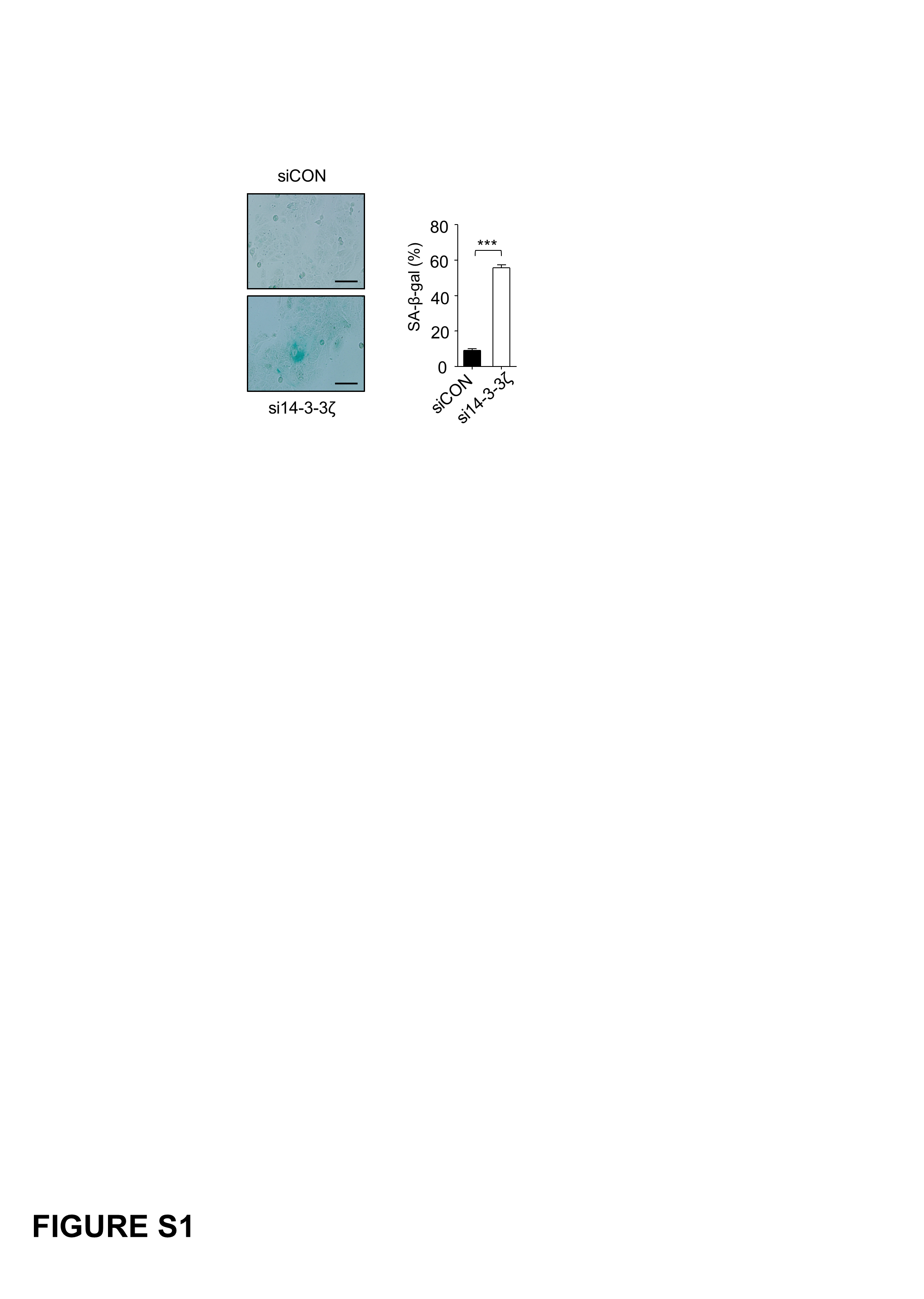

Supplement: Supplementary file 1 [file CPR-52-e12654-s001.TIF]

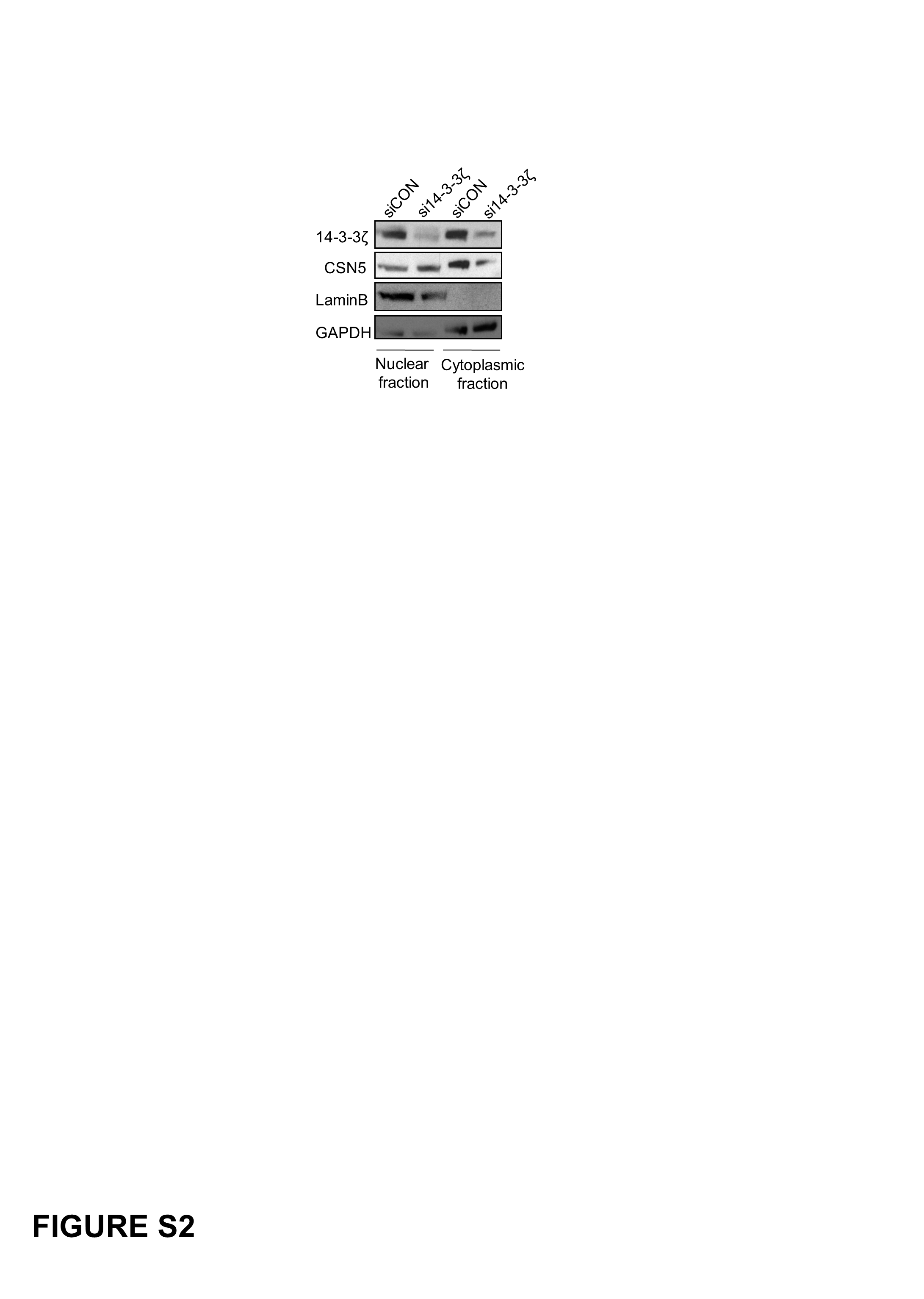

Supplement: Supplementary file 2 [file CPR-52-e12654-s002.TIF]
