# Supplementary material for: 14‐3‐3ζ targeting induced senescence in Hep‐2 laryngeal cancer cell through deneddylation of Cullin1 in the Skp1‐Cullin‐F‐box protein complex
Source: Cell Prolif. 2019 Jun 21;52(5):e12654. doi: 10.1111/cpr.12654 (PMC6797561; doi:10.1111/cpr.12654)
Supplement: Supplementary file 3 [file CPR-52-e12654-s003.docx]

**Supplementary Table 1.The sequence of siRNAs used in this study**

| Gene name | Sequence (5’→3’) |
| --- | --- |
| Control  14-3-3ζ  p27  Cdh1 | CCUACGCCACCAAUUUCGU  CGCUAAUAAUGCAAUUACUGA  AAGUACGAGUGGCAAGAGGUG  UGAGAAGUCUCCCAGUCAG |
| CSN5 | GCACUGAAACAAAUGAUUA |
|  |  |

| Gene name | Sequence (5’→3’) |
| --- | --- |
| 14-3-3ζ | F: CGATCAGTCACAACAAGCAT  R: AGCATGGATGACAAATGGTC |
| Skp2 | F: CCCACGGAAACGGCTGAAGA  R: CGCTAGGCGATACCACCTCTTACAA |
| p27  Cdh1  β-actin | F: TGCAACCGACGATTCTTCTACTCAA  R: CAAGCAGTGATGTATCTGATAAACAAGGA  F: GATCTCCAAGATCCCCTTCA  R: CCTCCAACATGGACAGCTTCT  F: AGTACTCCGTGTGGATCGGC  R: GCTGATCCACATCTGCTGGA |

**Supplementary Table 2. Primer sequences used in this study**
