# Supplementary material for: 14‐3‐3ζ targeting induced senescence in Hep‐2 laryngeal cancer cell through deneddylation of Cullin1 in the Skp1‐Cullin‐F‐box protein complex
Source: Cell Prolif. 2019 Jun 21;52(5):e12654. doi: 10.1111/cpr.12654 (PMC6797561; doi:10.1111/cpr.12654)
Supplement: Supplementary file 4 [file CPR-52-e12654-s004.docx]

Supplemental Figure Legends

Figure S1. 14-3-3ζ depletion induced senescence in SNU899 laryngeal cells.

SNU899 cells were transfected with14-3-3ζ siRNA for 3 days. Senescence induction was evaluated by SA-ß-gal staining (left) and presented as the percentage (%) of SA-ß-gal-positive cells (right). Scale bar, 100 µm. *** *P*< 0.001.

Figure S2. Subcellular distribution of CSN5 following depletion of 14-3-3ζ in Hep-2 cells.

Hep-2 cells were treated with14-3-3ζ siRNA for 3 days and subjected to subcellular fractionation. The cytoplasmic fraction was extracted using CE buffer (HEPES 10 mM pH 7.9, KCl 10 mM, EDTA 0.1 mM, NP-40 0.3%) after centrifugation at 2800 x g for 2 min. The pellet (nuclear fraction) was resolved using RIPA buffer. After determination of protein concentration, 4 times more proteins than the cytoplasm were loaded for nuclear fraction for western blotting of CSN5. Lamin B and GAPDH were used as fractionation markers for the cytoplasmic and nuclear fractions, respectively, as well as a loading control.
